# Stammering

**Published:** 1858-09

**Authors:** 


### STAMMERING.

Is sometimes the result of habit or carelessness, at others, it succeeds a long attack of sickness. It is a kind of St. Vitus' Dance of the tongue. Not unfrequently it is brought on by the harsh treatment or inveterate ill-nature of parents, teachers, or superiors, in habitually meeting those under them with threatenings, scolding, or fault-finding. We have met before now with a most miserable class of human, or rather inhuman beings, who scarcely ever enter a room, where are children, or servants, or dependants, without the expression of some disapprobation or complaint. This has very naturally the effect to confuse and intimidate a child, especially one of a highly nervous or excitable temperament: while steadiness and composure are the very antipodes of stuttering, which is essentially the throwing out too much nervous power, sending too much nervous influence to the muscles which are employed in speaking; the result is, a want of proper control of those muscles. Hence, whatever diminishes the nervous supply to those parts, whatever directs the nervous flow to some other part of the body, diminishes the stammering in the same proportion. This is the principle of cure in all cases, although we have never seen a reference to it by any writer. Some twenty years ago, the New York world was struck with dumb amazement at the instantaneous remedy for stammering, which was thrusting a knitting-needle through the tongue. But it cured only until the tongue got well, because, while the tongue was sore from the barbarous operation, the extra nervous energy was expended in the instinctive effort to refrain from any other than a careful movement of the tongue. The expedient of Demosthenes in speaking with little pebbles in his mouth, was in the same direction. One of the most inveterate stammerers in London became possessed with a fancy that he would make a good actor. On his first appearance, the theatre was crowded, in curiosity. During the whole play, he did not mispronounce a single word, did not fail to utter distinctly a single syllable, because the mind was engaged in another effort, was excited in another direction, the extra nervous power found vent in another outlet—precisely as in the more recently alleged accidental discovery of a lady, that reading or speaking in a whisper is an instantaneous remedy; because it requires an effort to whisper, the mind's attention is directed to the act of whispering, and not to the distinctness of utterance. We will venture the assertion, that no man ever stammered in "popping the question," nor a young lady halt out y-ye-ye-yes. Instinct itself prompts a cure. After a long illness from an accident, our Robert, aged three years, suddenly began to stammer most vexatiously. His whole system was in a debilitated and irritable condition. He had never come in contact with a stammerer. And believing that scolding, or threats, or ridicule, would only serve to fix the habit for life, which would have been a great misfortune, we made an effort, without apparent effort, to divert his attention to some other thing than the stammering. For example, when he asked for anything, he was told: "Now, if you ask for it plainly, you shall have it;" and before we were aware of it, we found him, whenever he attempted to ask for anything, striking his little hand against his thigh, as he stood before us, at the enunciation of every syllable; and by encouragement, we found the habit broken up in a few months. As it is a life-long calamity to have a son or daughter grow up a stutterer, we trust these hints may be turned to practical account by those whom it may concern. Anything else done at the time of uttering each syllable, divides the attention, gives two outlets to the extra nervous flow, and the remedy is complete; make a mark, pull a string, turn a leaf, stamp the foot—any one of them will effect a cure in a reasonable time.

